# Prevalence and distribution of late gadolinium enhancement in a large population of patients with Duchenne muscular dystrophy: effect of age and left ventricular systolic function

**DOI:** 10.1186/1532-429X-15-107

**Published:** 2013-12-21

**Authors:** Kan N Hor, Michael D Taylor, Hussein R Al-Khalidi, Linda H Cripe, Subha V Raman, John L Jefferies, Robert O’Donnell, D Woodrow Benson, Wojciech Mazur

**Affiliations:** 1 Nationwide Children’s Hospital, Columbus, OH, USA; 2 Cincinnati Children’s Hospital, Cincinnati, OH, USA; 3 Duke University School of Medicine, Durham, NC, USA; 4 Ohio State University, Columbus, OH, USA; 5 University of Cincinnati, Cincinnati, OH, USA; 6 Children’s Hospital of Wisconsin, Milwaukee, WI, USA; 7 The Heart and Vascular Center at the Christ Hospital, Cincinnati, OH, USA

**Keywords:** Cardiovascular magnetic resonance, Duchenne muscular dystrophy, Late gadolinium enhancement, Ejection fraction, Myocardial fibrosis

## Abstract

**Background:**

Duchenne muscular dystrophy (DMD), an X-linked disorder affects approximately 1 in 5000 males, is universally associated with heart disease. We previously identified myocardial disease by late gadolinium enhancement (LGE) in DMD subjects at various stages of disease, but the true prevalence is unclear. Cardiovascular magnetic resonance (CMR) is well established for both assessment of ventricular function and myocardial fibrosis by LGE. We sought to establish i) prevalence and distribution of LGE in a large DMD population and ii) relationship among LGE, age, LVEF by CMR and current living status.

**Methods:**

Current living status, demographic and CMR data including ventricular volumes, LVEF and LGE from 314 DMD patients undergoing evaluation at a single large tertiary referral center were analyzed.

**Results:**

113 of 314 (36%) of DMD subjects showed LGE positivity with prevalence increasing from 17% of patients <10 years to 34% of those aged 10–15 years and 59% of those >15 years-old. Patients with LVEF ≥55% were LGE positive in 30% of cases; this increased to 84% for LVEF <55%. LGE was more prevalent in the free wall (531/1243, 42.7%) vs. septal segments (30/565, 5.3%). Patients with septal involvement were significantly older and had lower LVEF than those with isolated free wall LGE. Ten percent (11/113) patients who had LGE died 10.8 months after CMR. Only one patient from the LGE negative group died. Patients who died had higher heart rate, larger left ventricular volume and mass, greater number of positive LGE segment and increase incident of septal LGE compared to those who remained alive.

**Conclusion:**

In DMD patients, LGE occurs early, is progressive and increases with both age and decreasing LVEF. Segmentally, the incidence of the number of positive LGE segments increase with age and lower LVEF. Older patients and those who died during the study period had more septal LGE involvement. The current studies suggest that the time course and distribution of LGE-positivity may be an important clinical biomarker to aid in the management of DMD-associated cardiac disease.

## Background

Duchenne muscular dystrophy (DMD), an X-linked recessive disorder affecting approximately 1 in 5000 males is the most common inherited muscular dystrophy [1-3]. The disease results from mutations in the gene for dystrophin, a sarcolemmal protein, that is abundant in both cardiac and skeletal muscle [4]. Typically, progressive skeletal muscle weakness results in loss of ambulation between 7 and 13 years of age [5,6]. Corticosteroids and supportive respiratory devices [7-11] have improved motor and respiratory outcomes, resulting in DMD-associated cardiac disease as the leading cause of death typically in the second to third decade of life [8,12,13]. DMD-associated cardiac disease is progressive and ultimately results in global ventricular systolic dysfunction, often with minimal ventricular dilation [14]. End-stage cardiac pathology includes cardiomyocyte hypertrophy, atrophy and fibrosis [15-17].

Fibrosis of the left ventricle in DMD has been observed at autopsy [15,17] and during cardiovascular magnetic resonance (CMR) with late gadolinium enhancement (LGE) [18,19]. LGE appears to be associated with late stages of the disease [19] but the true prevalence of LGE and its relationship to disease state, e.g. age at CMR and left ventricular ejection fraction (LVEF), is uncertain. The purpose of the current study was to establish the prevalence of LGE across a large DMD population with broad age range and to correlate LGE with severity of DMD associated cardiac disease as characterized by LVEF and living status.

## Methods

### Study population

Current living status as of December 2012 and demographic data were analyzed from records of DMD patients who underwent clinical CMR studies including LGE between September 2005 and September 2012 at a single large tertiary referral center. Only patients with a known diagnosis of DMD confirmed by a skeletal muscle biopsy showing absent dystrophin and/or DNA analysis demonstrating a characteristic dystrophin mutation in all patients. The Institutional Review Board at Cincinnati Children’s Hospital approved the study.

### Cardiac magnetic resonance imaging protocols and data analysis

CMR was conducted either on a Siemens 3 Tesla Trio (Siemens Medical Solutions, Malvern, PA/Erlangen, Germany), Philips 3 Tesla Achieva (Philips Healthcare, Andover, MA) or on a 1.5 Tesla GE Signa Excite (General Electric Healthcare; Milwaukee, WI). Machine type was based solely on clinical availability, independent of the patient’s clinical status. Cardiac functional imaging was performed using a standard retrospective ECG-gated, segmented steady state free precession (SSFP) technique and includes a short axis stack of cine SSFP images from cardiac base to apex as previously described [20,21]. Typical scan parameters included FOV = 32–38 cm, slice thickness = 5–6 mm, NEX = 2 (breath hold; 4–5 for free breathing), TE/TR = 1.4/2.8 (Siemens), TE/TR = 2.0/4.0 (GE), in-plane resolution = 1.2– 2.2 mm. A minimum of 12 slices were performed. The typical temporal resolution of the cine SSFP images was 30–40 ms and was adjusted according to the patient’s heart rate and ability to breath-hold. The RF flip angles were set between 50°–70° dependent on the patient weight, height and the SAR level. Left ventricular volumes, mass and LVEF were assessed via standard planimetry techniques using semi-automated computer software (QMASS v.6.1.5, Medis Medical Imaging Systems, Netherlands) [20,21]. LGE status, ventricular volumes, mass, and EF along with subject demographic data were tabulated for each subject, and then exported to a spreadsheet file for off-line analysis.

**Figure 1:**
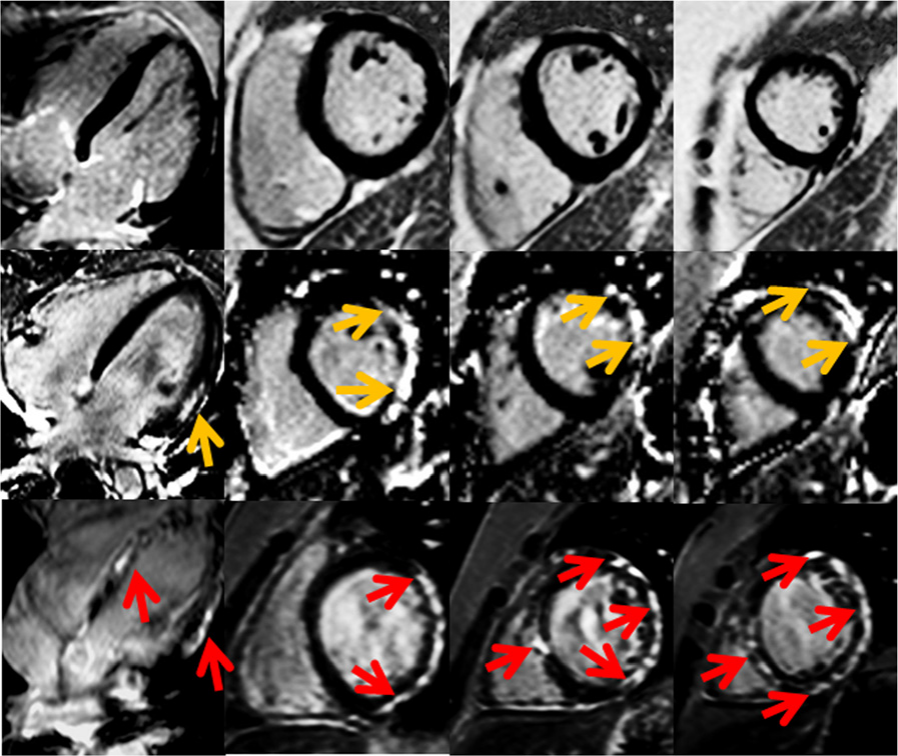
**Examples of LGE by CMR.** (Top Panel) 12 year old DMD patient with no LGE (dark myocardium), (Middle Panel) 8.5 year old DMD patient with LGE in the free wall only (bright areas shown by yellow arrows) and (Bottom Panel) 18 year old DMD patient LGE involving multiple segments including the septum (bright areas shown by red arrows).

LGE imaging was performed via a FLASH inversion sequence recovery protocol 5–8 minutes after 0.2 mmol/kg) gadolinium-based contrast agent injection as previously described [22,23]. The LGE sequence was independently analyzed by a single expert reader (KNH) blinded to the clinical report. LGE was deemed negative or positive globally at the base, mid-ventricle and apex as well as in each of 16 myocardial segments by visual rating [24] (Figure 1). At our center it is a standard of practice to report the presence of LGE using the modified 16-segment model which is exactly the same method we used for this study. The primary reader (KNH) independently reviewed all CMR studies for the presence of LGE without knowing the LGE status of the clinical report. For intra- and interobserver variability, the clinical LGE description from a subset of 60 randomly selected CMR studies from each of the primary reader (KNH) and a second CMR cardiologist (MDT) (30 LGE negative and 30 LGE positive studies) were reviewed.

### Statistical methods

Study results are expressed as mean ± SD for continuous data and as percentages and numbers for categorical data. Continuous variables were compared using two-sample t-test and categorical variables were compared using Fisher exact-test. LGE data were classified as being positive or negative and analyzed using a logistic regression model to estimate the odds ratio (OR) and 95% confidence interval (CI) between the patient aged: 10–15 years and > 15 years as compared to the reference age group of < 10 years. Similar model was used to estimate the OR (95% CI) between the LVEF of subjects with LVEF < 55% compared to those with LVEF ≥ 55%. Segments were summarized across the above age and LVEF groups. All tests were 2-sided, and a p-value < 0.05 was considered statistically significant. SAS version 9.2 (SAS Institute Inc., Cary, NC) was used for all analyses.

## Results

### Patient stratification

A total of 330 males, ages 6 to 28 years, with DMD underwent clinical CMR evaluation during the study period (Table 1). 16 subjects were excluded due a lack of intravenous access. Patients were dichotomized as LGE negative and LGE positive if any LV myocardial segment showed LGE positivity; 113/314 (36%) subjects were deemed LGE positive. LGE was always distributed in the sub-epicardial region and spares the sub-endocardium region. LGE-negative patients were younger than LGE positive patients (11.8 ± 3.4 vs. 15.2±5.1 years, p < 0.0001). Heart rate did not differ between the two groups but height; weight and BSA were higher in the LGE positive patients as expected based on older age. LVEF was lower and indexed left ventricular end-diastolic volume (LVEDV/BSA) and mass (LVM/BSA) were significantly larger for LGE positive compared to LGE negative patients suggesting more advanced heart disease (Table 1). Of the patients that were LGE positive, 11/113 (10%) died during the study period. Patients who died during the study period were older (19.5 ± 5.9 vs 14.7 ± 4.8 years, p = 0.003), had higher heart rates (110 ± 21 vs 96 ± 13.9 bpm, p = 0.003), larger ventricular volume (128.2 ±46.2 vs 73.1 ± 16.9 mL/m^2^, p < 0.0001), ventricular mass (69 ± 11.8 vs 51 ± 12.5 g/m^2^, p < 0.0001), lower LVEF (32 ±13.9 vs 59.7 ± 7.5 percent, p < 0.0001) and greater number of positive LGE segments (9.6 ± 4.2 vs 4.5 ± 2.4, p < 0.0001) compared to those who remained alive (Table 2). When compared to the clinical reports of the 60 randomly selected CMR reports from the primary reader ( KNH) and 60 clinical reports from a second cardiologist (MDT), there was 100% agreement with the primary reader’s (KNH) clinical LGE findings and the LGE findings later performed for the study. Likewise, there was 100% agreement between the clinical LGE findings of the second cardiologist (MDT) and the LGE findings later performed by the primary reader (KNH).

**Table 1:** Demographic and CMR findings between LGE negative and LGE positive patient groups

**Patient groups**	**LGE negative (n = 201)**	**LGE positive (n = 113)**	**P-value**
Age (years)	11.8 ± 3.4 (6–28)	15.2 ± 5.1 (7–32)	<0.0001
Heart rate (bpm)	100.8 ± 14.1 (55–143)	98 ± 15.7 (48–149)	0.056
BSA (m^2^)	1.2 ± 0.33 (0.8-2.6)	1.4 ± 0.3 (0.9-2.4)	<0.0001
Height (cm)	135.5 ± 16.9 (108–191)	147.3 ± 16.8 (117–191)	0.0004
Weight (kg)	42 ± 19.2 (19–136)	52 ± 18.1 (24–106)	<0.0001
LVEF (%)	64.8 ± 5.4 (35–78)	57 ± 11.6 (17–79)	<0.0001
LVEDV/BSA (mL/m2)	67.9 ± 13.9 (31–107)	76.9 ± 26.4 (35–207)	0.0004
LVM/BSA (g/m2)	46.3 ± 9.9 (24–78)	51.7 ± 13.2 (29–109)	<0.0001

*Abbreviations*: *BPM* Beat Per Minute, *BSA* Body Surface Area, *CM* Centimeter, *CMR* Cardiac Magnetic Resonance Imaging, *DMD* Duchenne Muscular Dystrophy, *g* gram, *kg* Kilogram, *LGE* Late Gadolinium Enhancement, *LVEF* Left Ventricular Ejection Fraction, *LVEDV* Left Ventricular End-Diastolic Volume, *LVM* Left Ventricular Mass, *mL* Milliliter, *m*^2^ Meter Square, *n* Number of patients.

**Table 2:** CMR findings and living status in DMD patients

**Patient groups**	**Alive (n = 102)**	**Not alive (n = 11)**	**P-value**
Age (years)	14.7 ± 4.8^*^	19.5 ± 5.9	0.003
Heart rate (bmp)	96 ± 13.9^*^	110 ± 21	0.003
CMR to death (months)	N/A	10.8 ± 8.5	N/A
LVEF (%)	59.7 ± 7.5^*^	32 ±13.9	<0.0001
LVEDV/BSA (mL/m^2^)	73.1 ± 16.9^*^	128.2 ±46.2	<0.0001
LVM/BSA (g/m^2^)	51 ± 12.5^*^	69 ± 11.8	< 0.0001
NPS with LGE	4.5 ± 2.4^*^	9.6 ± 4.2	<0.0001
Segments with LGE (%)	455/1632 (28%)	106/176 (60%)	N/A
Septal LGE (%)	13/510 (2.5%)	17/55 (31%)	N/A

*Abbreviations*: *BMP* Beats per minute, *CMR* Cardiac Magnetic Resonance Imaging, *DMD* Duchenne Muscular Dystrophy, *LGE* Late Gadolinium Enhancement, *LVEF* Left Ventricular Ejection Fraction, *LVEDV/BSA* Left Ventricular End-Diastolic Volume Indexed to Body Surface Area, *LVM/BSA* Left Ventricular Mass Indexed to Body Surface Area, *g/m*^2^ Grams Per Meter Square, *mL* Milliliter, *NPS* Number of Positive Segments, *Indicates Statistical Significance.

Patients were stratified into groups based on age (<10 years old, 10–15 years old and >15 years) and normal LVEF (≥ 55%) or reduced LVEF (< 55%) (Table 3). Among patients <10 years old 17% were LGE positive, this increased to 34% for those 10–15 year old and to 59% for those > 15 year old. Age was very strongly associated with presence of LGE with an odds ratio of 2.6 (age 10–15 years) and 7 (age > 15 years). In patients with LVEF ≥55% LGE positivity was seen in 30% but with LVEF < 55% this number increased to 84%. LVEF was a powerful predictor of presence of LGE with an odds ratio of 12.3 for those with LVEF < 55% (Table 3).

**Table 3:** CMR Findings between LGE negative and LGE positive DMD patient compared to age and LVEF

**Parameters**	**# Patients**	**LGE negative**	**LGE positive**	**NPS with LGE**	**Odds ratio (95% CI)**	**P value**
Age < 10 years	83	69 (83%)	14 (17%)	4.4 ± 3.0	1.0	-------
Age 10–15 years	149	98 (66%)	52 (34%)	4.8 ± 2.5	2.6 (1.3-5.0)	< 0.006
Age > 15 years	82	34 (41%)	48 (59%)	5.9 ± 3.3	7.0 (3.4-14.3)	< 0.0001
LVEF ≥ 55%	277	195 (70%)	82 (30%)	4.1 ± 2.4	1.0	-------
LVEF < 55%	37	6 (16%)	31 (84%)	7.8 ± 3.4	12.3 (4.9-30.6)	< 0.0001

*Abbreviations*: *#* Number, *CI* Confidence Interval, *CMR* Cardiac Magnetic Resonance Imaging, *DMD* Duchenne Muscular Dystrophy, *LGE* Late Gadolinium Enhancement, *LVEF* Left Ventricular Ejection Fraction, *NPS* Number of Positive Segments.

A multivariable model with LVEF (<55 versus ≥55 percent) as the main clinical outcome using a logistic model was performed. An un-adjusted model with +/-LGE in the model as a covariate which resulted in estimated OR for +/-LGE of 12.3 (95% CI 4.9, 30.6; p < 0.0001 and c-statistic = 77%) in predicting reduced EF was performed. Then age (as continuous variable) was added into the model resulted in an adjusted OR for +/-LGE of 7.6 (95% CI 2.9, 19.7; p < 0.0001 and c-statistic = 85%) in predicting reduced EF. Similar analysis using +/-septal LGE as a covariate in the model and LVEF as the main clinical outcome was conducted and this resulted in un-adjusted estimated OR for +/-septal LGE of 7.0 (95% CI 2.3, 21.1; p = 0.0006 and c-statistic = 64%) in predicting reduced EF. Then age (as continuous variable) was added into the model with resulted in an adjusted OR for +/-septal LGE of 5.2 (95% CI 1.6, 16.5; p = 0.0059 and c-statistic = 78%) in predicting reduced EF.

### LGE has a regional distribution

**Figure 2:**
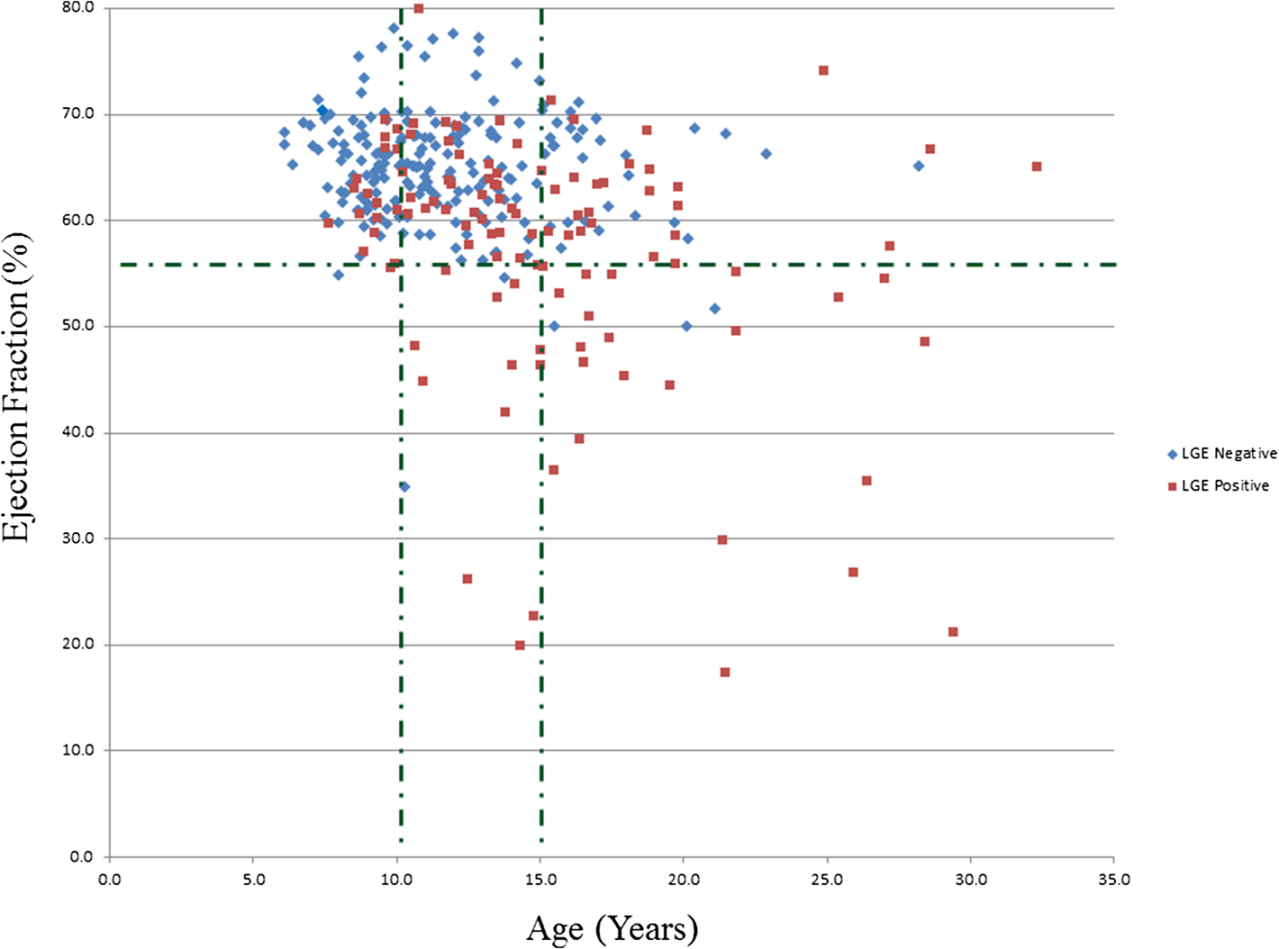
**Scatter graph of LVEF versus age.** The LVEF of LGE negative (blue diamonds) and LGE positive (red square) patients are plotted against age demonstrating LGE was associated with older age and lower LVEF.

**Figure 3:**
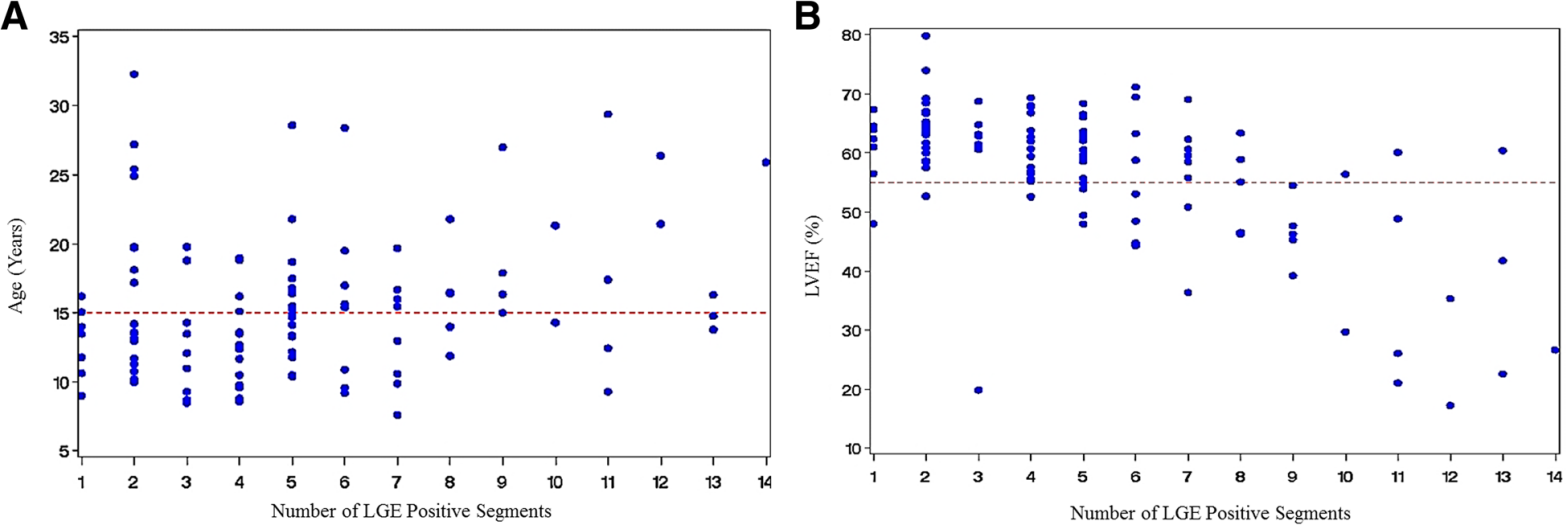
**Scatter plot of number of LGE positive segment versus age and LVEF.** Scatter Plot of the Number of LGE positive segment compared to age **(A)** and LVEF **(B)**. The number of LGE positive segments was associated older age and lower LVEF.

**Figure 4:**
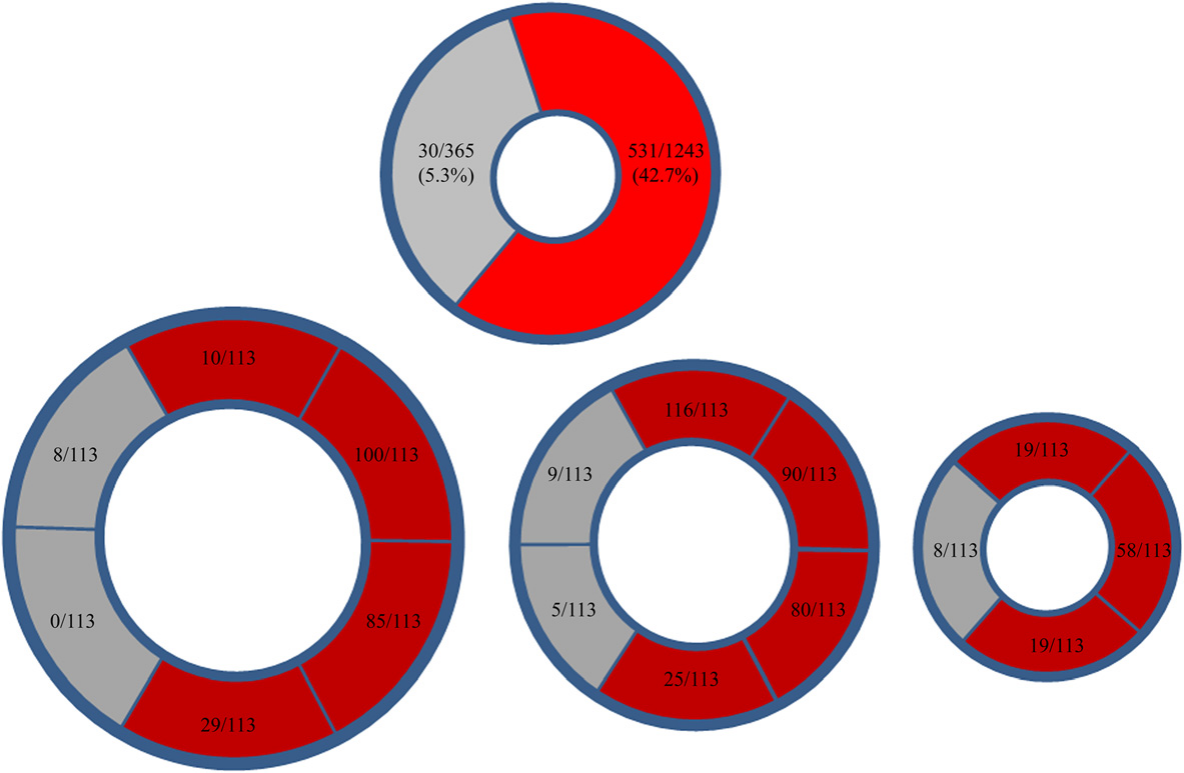
**Global and segmental LGE. (A)** LGE was more prevalent in the free wall segments (red) compared to the septal segments (gray) 42.7% (531/1243) versus 5.3% (30/565). **(B)** Segmentally, free wall segments (red) were more commonly affected with LGE than the septal segments (gray).

Figure 2 shows a plot of age and LVEF for each patient (LGE-negative shown in blue and LGE-positive shown in red). To quantify a segmental analysis of LGE, we analyzed studies of 113 patients that were LGE positive. The number of LGE positive segments was associated older age and lower LVEF (Figure 3A-B). Of the 1808 segments analyzed, 565 were septal segments and 1243 were free wall segments. For each CMR study, the number of LGE positive segments ranged from 1 to 13 (Figure 3A-B). In subjects < 10 years of age, mean number of LGE positive segments was 4.4±3.0. This increased to 4.8±2.5 for subjects 10–15 years of age and 5.9±3.3 LGE positive segments for subjects >15 years old. For subjects with LVEF ≥ 55%, mean number of LGE positive segments was 4.1±2.4 versus 7.8±3.4 LGE positive segment for LVEF > 55% (Table 3). Overall, LGE was more prevalent in the free wall segments compared to the septal segments 42.7% (531/1243) versus 5.3% (30/565). At the base and mid-ventricle LGE was most prevalent in anterolateral (n = 190), inferolateral (n = 165) and inferior segments (n = 54) and less common in the anterior segment (n = 26). Of the septal segments, the anteroseptal segment (n = 17) was more commonly affected than the inferoseptal segment (n = 5). At the apical level, the findings are similar with the lateral segments (n = 58) most commonly affected compared to the inferior (n = 19) and anterior (n = 19) segments and apical septal segment least affected (n = 8) (Figure 4).

### Predictors of Septal vs. Isolated Free Wall LGE

Septal LGE-positivity was never found in isolation and only in association with LGE positivity in other free wall segments. Patients with septal LGE involvement were older than those without septal LGE involvement (18.3 ± 5.5 vs 14.6 ± 4.9, p =0.006), though heart rate and BSA were not statistically different between the two groups. Among patients age < 10 years (n = 14) the incidence of septal LGE was 7.1% (95% CI, 0.18 – 33.8), for those age 10–15 years (n = 51) the incidence of septal LGE of 7.8% (95% CI, 2.2-18.9) and increased to 25% (95% CI, 13.6 – 39.6) for patients > 15 years of age (n = 48) (Table 4). The number of total positive LGE segments was greater when LGE was evident in the septum (4.2 ± 2.2 versus 9.4 ± 3.1, p < 0.0001). Furthermore, septal LGE was associated with greater indexed left ventricular end-diastolic volume (111.2 ± 44.1 versus 72.7 ± 17.1, p < 0.0001) and left ventricular mass (61.1 ± 15.2 versus 51.3 ± 12.7, p = 0.006) as well as lower LVEF (42.6 ± 16.9 versus 59.5 ± 8.3, p < 0.0001) at the time of CMR (Table 4). Patients who died during the study period not only have greater number of LGE positive segments, the percent of segments with LGE was higher with 106/176 (60%) vs455/1632 (28%) and had more septal involvement 17/55 (31%) vs 13/510 (2.5%) compared to those who remained alive during the study period (Table 2).

**Table 4:** CMR findings between patients with no septal LGE and those with septal LGE involvement

**Patient groups**	**No septal involvement (n = 96)**	**Septal involvement (n = 17)**	**P-value**
Age (years)	14.6 ± 4.9^*^	18.3 ± 5.5	0.006
Heart rate (bmp)	97.5 ± 16.7	97.3 ± 7.9	0.97
BSA (m^2^)	1.4 ± 0.32	1.5 ± 0.29	0.22
LVEF (%)	59.5 ± 8.3^*^	42.6 ±16.9	<0.0001
LVEDV/BSA (mL/m^2^)	72.7 ± 17.1^*^	111.2 ±44.1	<0.0001
LVM/BSA	51.3 ± 12.7^*^	61.1 ± 15.2	0.006
NPS with LGE	4.2 ± 2.2^*^	9.4 ± 3.1	<0.0001
Age < 10 years (n = 14)	--------------	7.1% (95% CI, 0.18-33.9)	0.001
Age 10 – 15 years (n = 51)	--------------	7.8% (95% CI, 2.2-18.9)	<0.0001
Age > 15 years ( n = 48)	--------------	25.0% (95% CI, 13.6-39.6)	0.0005

*Abbreviations*: *BSA* Body Surface Area, *BMP* Beats per minute, *CI* Confidence Interval, *CMR* Cardiovascular Magnetic Resonance, *LGE* Late Gadolinium Enhancement, *LVEF* Left Ventricular Ejection Fraction, *LVEDV* Left Ventricular End-Diastolic Volume, *LVM* Left Ventricular Mass, *mL* Milliliter, *m*^2^ Meter Square, *n* Number of patients, *NPS* Number of Positive Segments, * = P value (<0.05) is significant.

## Discussion

This is the first study to establish prevalence and distribution of LGE in a large DMD population and to establish the relationship to age and LVEF. The major finding of this study is that in DMD patients, LGE-positivity, an established indicator of injured or fibrotic myocardium, is more prevalent with increasing age and decreasing LVEF. This is not unexpected as observations in other forms of heart disease, e.g. ischemic, hypertrophic and dilated cardiomyopathies as well as aortic valve disease, suggest that LGE-positivity is associated with worse outcomes, likely representing common end stage process regardless of underlying disease pathogenesis [25-33]. In addition, the cohort of patients that died during the study period not only have greater number of positive LGE segments but greater percent of septal LGE involvement associated with increase heart rate, left ventricular volume, mass and ejection fraction. Further, findings in the patients we studied suggest that the distribution of LGE, i.e. free wall only vs free wall plus septal may be indicative of heart disease severity.

The presence of LGE was initially reported in 8 boys with DMD by Silva et al. [18]. Subsequently, Puchalski et al. reported 74 patients with DMD, 32% had LGE involving the posterobasal region of the LV in a subepicardial distribution [19]; they reported that more advanced DMD-associated heart disease correlated to the presence of LGE in the inferior and left lateral free wall with transmural fibrous replacement. Results of the current study confirm that boys with LGE-positivity were significantly older and had lower LVEF than those without LGE. However, we report for the first time an association of global and segmental LGE with age and EF. The youngest DMD patient in our cohort to have LGE was 7.6 years age despite normal LVEF which indicates that DMD-associated heart disease in the form of LGE can occur before age 10 years of age. Walcher et al. analyzed a small number of female carriers and affected males (7 patients in total) and concluded that LGE is present prior to the onset of global left ventricular dysfunction [34]. Bilchick et al. evaluated the prevalence and distribution of regional scar on dysfunctional myocardial segments in a small DMD patient population (16 patients) and concluded that overall scar prevalence in inferior, inferolateral and anterolateral segments was eight times higher than in inferoseptal, anteroseptal and anterior segments [35,36]. Our study demonstrated a similar distribution of LGE but in a considerably larger cohort, notably the largest, of DMD CMR exams.

The finding of LGE, a measure of cardiac fibrosis and progressive cardiac disease in DMD boys, is intuitively expected but the natural history has been poorly characterized with regards to the age of onset and association with LV systolic dysfunction. DMD results from mutation in the gene for the protein dystrophin. Dystrophin provides a structural link between the cytoskeleton and the extracellular matrix, and mutations result in greatly reduced or absent dystrophin resulting in a loss of cell membrane integrity. A long-standing hypothesis regarding DMD disease pathogenesis implicates loss of membrane integrity as a primary event leading to degeneration of myocytes. Intermittent tears in the cell membrane permit influx of calcium that leads to a destructive cascade culminating in myocyte necrosis, inflammation, and fibrosis [37-39]. These processes are ongoing in early stages of disease, but a cumulative effect is required for clinical detection by LGE using CMR.

While the mechanism of myocardial injury in DMD remains somewhat speculative, Mavrogeni et al. reported on a population of 20 patients with DMD and found that6 patients were LGE positive; LGE positive patient, 4 had CMR (STIR-T2 weighted imaging) evidence of myocarditis. All 6 patients with LGE had histologic evidence of myocarditis and rapid deterioration of LVEF was noted in those patients in 1 year follow up CMR [40,41]. Wansapura et al. demonstrated increase in the heterogeneity of T2 signal with increasing age and decreasing LVEF, likely representing micro fibrosis (shortening T2) coexisting with tissue edema (prolonging T2) [42]. In a novel therapeutic approach to DMD in a murine DMD model, Rafael-Fortney et al. showed marked reduction in myocardial fibrosis with the use of lisinopril and spironolactone [43]. The ability to quantify myocardial fibrosis noninvasively in humans using LGE-CMR suggests it may be a useful biomarker endpoint for therapeutic clinical trials.

### Study limitations

This was a retrospective study and as a result is subject to accepted limitations of this design. There is no correlation with LGE findings with medications such time on steroid and ACE inhibition, however patients are typically on steroid by age 5 years and typically remain on the steroid regimen for life. ACE inhibition is typically added by age 10 years or when there are signs of left ventricular dysfunction is evident by echocardiogram and independent of LGE findings. Although we have mortality data the numbers were small and since all patients died out of the hospital, we do not have the cause of death. The purpose was to define the natural history of LGE presence in the DMD population; future studies should focus on correlations with clinical outcomes (such as hospitalization and heart failure classification) and genotype-phenotype correlations. It is acknowledged that this would be valuable information and warrants additional future investigation. In addition, the data was accrued from a single center which could be seen as a limitation or a strength as it facilitated consistent image acquisition and interpretation. Only qualitative assessment of LGE is available for this study. We found it difficult to perform quantitative assessment of LGE in our patient population using either threshold or full width at half maximum (FWHM) methods, nature of the disease and epicardial LGE location bordering on epicardial fat compared to those reported in ischemic heart disease. T1 mapping, an emerging technique for characterization of the myocardial extracellular space, was not used in this work and warrants ongoing evaluation in DMD cardiomyopathy [44].

## Conclusions

This study documents evidence of scar burden at an age earlier than has been previously described for DMD-associated cardiac disease [45]. As such, these findings alter our understanding of DMD cardiac manifestations as it was previously felt that the myocardium was rarely affected by fibrosis prior to the age of 10 years [10,11]. Evaluation of the myocardium by CMR to document the presence of LGE may have important implications for the ongoing management of boys with DMD. We speculate that in future studies, LGE may prove to be a useful biomarker and could serve as the outcome of therapeutic strategies to assess utility of antifibrotic agents such as spironolactone or eplerenone to alter the natural history of DMD-associated cardiac disease [43,46,47].

## Abbreviations

DMD: Duchenne muscular dystrophy; BSA: Body surface area; CMR: Cardiovascular magnetic resonance; LVEDV: Left ventricular end-diastolic volume; LVEF: Left ventricular ejection fraction; LGE: Late gadolinium enhancement; SSFP: Steady state free precession; OR: Odds ratio; CI: Confidence interval.

## Competing interests

The authors declare that they have no competing interests.

## Authors’ contributions

KNH, WM and DWB contributed to all aspects of the manuscript conception, design, data analysis, collection, critical revision and final approval. MDT, SVR and LHC participated in the design and coordination of the study and helped to draft the manuscript. HRA provided statistical support and helped to draft the manuscript. JLJ and ROD helped draft, reviewed and revised the manuscript. All authors read and approved the final manuscript.
